# Impact of Fitness on Cardiac Torsion and Wall Mechanics in Ischemic Heart Disease Study (FIT-TWIST)

**DOI:** 10.3390/jcdd13020062

**Published:** 2026-01-24

**Authors:** Priscilla Wessly, Maiteder Larrauri Reyes, Syed I. Zaidi, Selin Sendil, Tarec K. Elajami, Christos G. Mihos

**Affiliations:** 1 Aurora Cardiovascular and Thoracic Services, Aurora Sinai/Aurora St. Luke’s Medical Centers, Milwaukee, WI 53215, USA; pwessly7@gmail.com; 2 Echocardiography & Non-Invasive Cardiovascular Laboratory, Division of Cardiology, Mount Sinai Medical Center, DeHirsch Meyer Tower 1st Floor, 4300 Alton Road, Miami Beach, FL 33140, USA; maiteder.larraurireyes@msmc.com (M.L.R.); selinsendil@gmail.com (S.S.); tarec.elajami@msmc.com (T.K.E.); 3 Orlando Health Heart and Vascular Institute, Orlando, FL 32714, USA; simzed@gmail.com

**Keywords:** cardiac mechanics, cardiac rehabilitation, global longitudinal strain, ischemic heart disease, speckle-tracking, strain echocardiography

## Abstract

Background: Cardiac rehabilitation (CR) and mechanics are individually associated with cardiovascular outcomes in ischemic heart disease (IHD); however, their interaction remains less defined. We hypothesized that a 36-session CR program improves cardiac strain and torsional mechanics in IHD patients. Methods: Ninety IHD patients on guideline-directed medical therapy with complete revascularization were prospectively enrolled, of which 27 electively completed a 36-session standardized exercise CR program. Speckle-tracking echocardiography was utilized to assess left ventricular (LV) global longitudinal strain (GLS) and peak twist, and right ventricular free wall strain (RVFWS) at baseline and after program completion. Participants were propensity-scoring matched 1:1 with 27 patients who declined participation (No-CR). Results: Clinical characteristics were similar between groups (mean age: 63 ± 10 years, 82% male, 31% three-vessel coronary artery disease). When compared with baseline, the CR group experienced a significant improvement in LV GLS (−14.9 ± 2.9 vs. −16.2 ± 3.1%, *p* = 0.003), with a numerical but non-significant increase in peak LV twist (14.4 ± 7.4 vs. 16.8 ± 5.3°, *p* = 0.162). The No-CR group showed significant deterioration in RVFWS (−22.9 ± 4.6% vs. −19.3 ± 5.4%, *p* = 0.009), with no other changes including in GLS (−14.8 ± 3.1 vs. −15 ± 3.3%, *p* = 0.831). Follow-up comparisons between CR versus No-CR revealed significantly greater peak LV twist (16.8 ± 5.3 vs. 12.1 ± 4.2°, *p* = 0.001) and a healthier RVFWS (−22.2 ± 4.5 vs. −19.3 ± 5.4, *p* = 0.044) in CR participants. Conclusions: CR in patients with IHD improved LV GLS and, compared with No-CR, conferred better LV twist and RVFWS.

## 1. Introduction

Ischemic heart disease (IHD) remains a leading cause of morbidity and mortality worldwide, accounting for over 9 million deaths annually and contributing to significant cardiovascular complications, particularly in patient’s post-revascularization [1]. Despite advances in medical and interventional therapies, IHD patients are at risk of adverse cardiac remodeling, which results in heart failure and major adverse cardiovascular events [2,3]. Cardiac rehabilitation (CR) is a multidisciplinary, systematic intervention that applies supervised exercise regimens in conjunction with secondary prevention therapies to improve outcomes in IHD patients. Following myocardial infarction, CR has been shown to reduce cardiovascular mortality, decrease hospital admissions, and improve health-related quality of life [4,5]. Clinical guidelines strongly recommend comprehensive CR programs for patients with acute coronary syndromes, post-coronary artery bypass surgery, or post-percutaneous coronary intervention (Class I, Level of Evidence: A) [6].

The impact of CR on advanced cardiac functional parameters in IHD, such as myocardial deformation and rotational mechanics, remains underexplored despite their strong individual associations with outcomes and prognosis [7]. Prior studies on CR and cardiac mechanics have been heterogeneous in terms of the study population, CR protocols, and duration of training. Analyses have mainly focused on left ventricular (LV) global longitudinal strain (GLS), which has appeared to improve with shorter endurance training CR protocols after myocardial infarction, while equivocal findings are reported in studies utilizing high-intensity interval or mixed resistance training [8,9,10,11,12]. Within this context, it is notable that post-infarct revascularization and surveillance GLS measures are strongly predictive of adverse LV remodeling, major adverse cardiovascular outcomes, and survival [7].

We hypothesized that a 36-session CR program in IHD would confer improvements in left and right ventricle cardiac mechanics and sought to compare these parameters between patients completing CR with a cohort who did not participate (No-CR group). As such, the present study seeks to provide physiologic insight on the benefits of CR in IHD to support clinical guidelines and optimize CR program design for IHD patients.

## 2. Methods

### 2.1. Study Design

The ‘Impact of Fitness on Cardiac Torsion and Wall Mechanics in Ischemic Heart Disease Study (FIT-TWIST)’ was a prospective, longitudinal, cohort study designed to assess the effects of physical activity and exercise on cardiac mechanics in patients with IHD. The protocol was reviewed and approved by the Mount Sinai Medical Center Institutional Review Board (Miami Beach, FL, USA) according to the research guidelines set forth by the 1975 Declaration of Helsinki (revised in 2013), and all participants signed informed written consent. This study was supported by a research grant awarded by the Miami Heart Research Institute/Florida Heart Research Foundation (Miami, FL, USA). The ethical approval number for our study is FWA00000176.

Prior to beginning training, subjects underwent a cardiopulmonary exam; an ECG; a treadmill exercise stress echocardiogram utilizing the Bruce protocol to assess for myocardial ischemia and measure functional capacity; and a resting echocardiogram with measurements of cardiac geometry, function, and mechanics (strain and torsion). The training period lasted 3 months, at which time the same measurements performed at baseline were repeated in all participants, as well as an exercise stress echocardiogram utilizing the Bruce protocol in the CR patients.

### 2.2. Study Population

Patients with IHD who were admitted to Mount Sinai Medical Center between March 2019 and April 2023 were screened for eligibility and enrollment. The inclusion criteria were (1) ≥18 years of age; (2) history of myocardial infarction confirmed by clinical testing and coronary angiography with complete revascularization as recommended by the American College of Cardiology/American Heart Association guidelines; (3) maximally tolerated guideline-directed medical and device therapy; (4) willingness to participate in a structured exercise training regimen and have baseline and follow-up exercise stress echocardiograms; and (5) baseline functional capacity ≥ 4 metabolic equivalents of task (METs). The exclusion criteria were (1) significant valvular or structural heart disease; (2) history of ventricular tachyarrhythmia or uncontrolled atrial tachyarrhythmia; (3) significant pulmonary disease; (4) uncontrolled hypertension (>160/100 mmHg); and (5) completion of <80% of the CR program sessions. Individuals who consented and met inclusion criteria but elected not to participate in the exercise training protocol were enrolled in the No-CR group and were matched in a 1:1 fashion with the CR group.

### 2.3. Clinical Assessment and Data

The demographics and clinical risk factor assessments were performed, and data was collected at the index hospitalization of each patient. This included review of all angiograms, stratification of the coronary artery disease severity, identification of the coronary syndrome, and medical regimen reconciliation. Compliance with the guideline-directed medical therapy was confirmed at the appointments for the baseline and follow-up stress echocardiogram.

### 2.4. Cardiac Rehabilitation Program

The CR program consisted of 36 total sessions that were completed 3 times per week over a 12-week period. Training sessions were administered by a certified exercise physiologist in the outpatient setting with continuous hemodynamic monitoring and in accordance with American Heart Association guidelines and recommendations [6,13]. Each session comprised warm-up (5 min), exercise (30 to 40 min), and cool down (5 min) periods. The exercise consisted of active stretching and primary aerobic activity with the use of a treadmill or stationary bicycle—patients were additionally encouraged to perform calisthenic exercises if feasible and tolerated. Month one exercise periods were conducted within zones 0 to 1 (<70% of maximum predicted heart rate), month two introduced training that included periods of zone 2 (70 to 90% of maximum predicted heart rate) and occasional zone 3 intensity (≥90% of maximum predicted heart rate), and month three maximized exercise in zone 3.

### 2.5. Two-Dimensional Speckle-Tracking, and Stress Echocardiography

All transthoracic echocardiograms were performed using a GE e95 or s70 cardiovascular ultrasound system (General Electric Healthcare, Waukesha, WI, USA), with all images acquired at a frame rate of 40 to 70 frames/second over three cardiac cycles and analyzed off-line from stored cine loops. The assessment of cardiac geometry, function, and diastology were performed off-line using a dedicated software package (EchoPAC v206, General Electric Healthcare, Waukesha, WI, USA) and in accordance with the American Society of Echocardiography guidelines [14,15].

**Figure 1:**
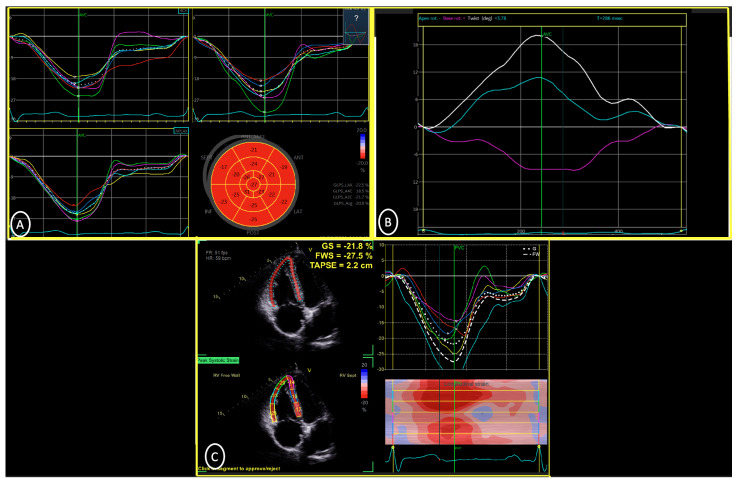
Cardiac mechanics assessed in the FIT-TWIST study. Examples of the cardiac mechanics assessments performed as part of the FIT-TWIST study. (**A**) LV GLS assessment depicted in segmental curves, a bullseye plot, and values. (**B**) LV twist and torsion, with apical (light blue), basal (purple), and net twist (white) depicted. (**C**) RV GLS and RVWS depicted in segmental curves, color M-mode, and values. 2CH = apical 2-chamber; 4CH = apical 4-chamber; AVC = aortic valve closure; AVG = average; GLS = global longitudinal strain; GLPS = global longitudinal peak strain; GS = global strain; LAX = apical long-axis; LV = left ventricle; ROT = rotation; RVFWS = right ventricular free wall strain; TAPSE = tricuspid annular plane systolic excursion.

Cardiac mechanics were analyzed using the two-dimensional speckle-tracking technique and included the following measures: (1) GLS (%)—maximal longitudinal shortening of the LV during the cardiac cycle measured in the apical four-, three-, and two-chamber views and averaged; (2) right ventricular free wall strain (RVFWS, %)—maximal longitudinal shortening of the RV free wall during the cardiac cycle measured in the RV-focused apical four-chamber view as the averaged basal, mid, and apical free wall segment values; (3) peak LV twist (degrees)—calculated by subtracting the short-axis LV basal rotation (negative value) from apical rotation (positive value), with the aortic valve closure time serving as a reference point for the end of systole, the LV base identified as the most proximal basal imaging plane with full circumferential wall thickness visualized at end-systole, and the LV apex identified as the most apical imaging window comprising clear endocardial borders and a circular LV geometry distal to the papillary muscles; (4) time to peak LV twist (ms)—time interval from the onset of systole to peak twist; and (5) time to peak LV untwist (ms)—time interval from the onset of systole to the nadir of LV twist (Figure 1) [7,16,17].

Stress echocardiography was performed utilizing a treadmill Bruce protocol with continuous blood pressure and electrocardiogram monitoring. The LV systolic function and wall motion were assessed at baseline (rest) and immediately following exercise (peak) in the parasternal long-axis, short-axis, and apical 4- and 2-chamber views. The presence of inducible myocardial ischemia was assessed as a new or worsening regional wall motion abnormality and reported within the recommended 17-segment model [18].

### 2.6. Statistical Analysis

Continuous variables were expressed as mean ± standard deviation (SD), and categorical variables were expressed as number and percentage. An independent Student’s *t*-test was utilized for intergroup comparisons, and a paired *t*-test for intragroup repeated measures. A chi-square or Fisher’s exact test was applied to categorical variables, as appropriate. Normality of continuous variables was assessed using the Shapiro–Wilk test and by visually inspecting the Q-Q plots. Patients in the No-CR group were matched with the CR group in a 1:1 fashion with a caliper width of 0.2 utilizing logistic regression analyses based on demographics (age and gender), severity of coronary artery disease (history of ST-elevation myocardial infarction, multi-vessel disease, and anterior myocardial infarction), LV function (ejection fraction and global longitudinal strain), and the availability of follow-up echocardiography. A multiple imputation model using multivariate linear regression was applied for missing-at-random continuous echocardiographic variables [19]. Statistical significance for all analyses was considered at *p* < 0.05. Statistical analyses were performed using SPSS, version 20 (IBM Statistical Product and Service Solutions, Chicago, IL, USA).

## 3. Results

### 3.1. Study Population Demographics and Clinical Characteristics

A total of 90 patients with IHD consented to and enrolled in this study, of which 27 electively completed the training program (CR group). Forty-six patients elected to not participate in exercise training. From this cohort, there were 27 who were propensity-matched in 1:1 fashion to the CR participants and represented the No-CR group. The remaining 17 patients were individuals who elected to participate in exercise training but completed < 80% of their CR sessions and were excluded from all analyses.

The mean age of the study population was 63 ± 10 years, and 82% were male. Co-morbidities included a history of smoking in 33%, hypertension in 74%, diabetes mellitus in 30%, and dyslipidemia 63%, with no difference in intergroup prevalence. Coronary artery disease profiles were similar, with three-vessel disease in 31% of patients, ST-elevation myocardial infarction in 43%, anterior wall myocardial infarction in 44%, and 76% of patients revascularized by percutaneous coronary intervention. Guideline-directed medical therapy was aggressively prescribed and balanced in both groups, with compliance confirmed at baseline and follow-up in all participants (Table 1).

### 3.2. Two-Dimensional and Speckle-Tracking Echocardiography

**Figure 2:**
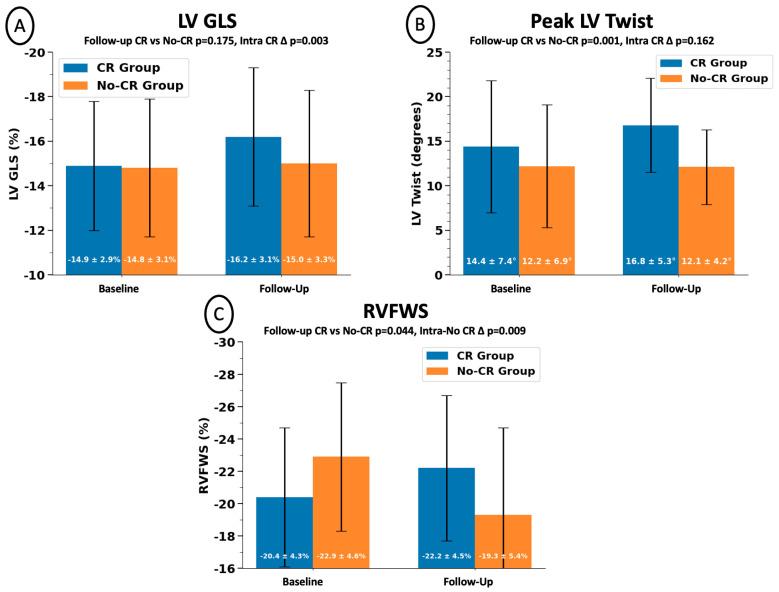
Cardiac mechanics in patients according to participation in cardiac rehabilitation. Changes in measures of cardiac mechanics in IHD patients enrolled in standardized 36-session CR versus those not participating in the FIT-TWIST study. (**A**) Improved LV GLS within the CR group (−14.9 ± 2.9% to −16.2 ± 3.1% (*p* = 0.003). (**B**) Higher follow-up peak LV twist in the CR versus No-CR groups (16.8 ± 5.3° vs. 12.1 ± 4.2°, *p* = 0.001). (**C**) Better follow-up RVFWS in the CR versus No-CR groups (−22.2 ± 4.5% vs. −19.3 ± 5.4%, *p* = 0.044), and interval worsening within No-CR. Data presented as mean ± standard deviation with pertinent *p*-values. CR = cardiac rehabilitation; GLS = global longitudinal strain; LV = left ventricle; RVFWS: right ventricular free wall strain; No-CR = no cardiac rehabilitation.

All parameters of cardiac geometry, function, and mechanics were similar between groups at baseline, except for an attenuated RVFWS in the CR group when compared with No-CR (−20.4 ± 4.3 vs. −22.9 ± 4.6%, *p* = 0.045). When compared with their baseline values, patients in the CR group experienced significant improvements in LVEF (52 ± 7 vs. 55 ± 8%, *p* = 0.010), LV GLS (−14.9 ± 2.9 vs. −16.2 ± 3.1%, *p* = 0.003), and E/e’ ratio (10 ± 3 vs. 9 ± 3, *p* = 0.032). A numerical increase in peak LV twist was also observed without reaching statistical significance (14.4 ± 7.4 vs. 16.8 ± 5.3°, *p* = 0.162). In the No-CR group, RVFWS significantly worsened from baseline (−22.9 ± 4.6% vs. −19.3 ± 5.4%, *p* = 0.009), without change in any other parameters. When comparing the CR versus No-CR groups at follow-up, those who participated in CR had a larger LV end-diastolic volume index (56 ± 17 vs. 47 ± 16 mL/m^2^, *p* = 0.040), greater peak LV twist (16.8 ± 5.3 vs. 12.1 ± 4.2°, *p* = 0.001), and better RV FWS (−22.2 ± 4.5 vs. −19.3 ± 5.4%, *p* = 0.044) (Table 2; Figure 2; Supplementary Tables S1 and S2).

### 3.3. Stress Echocardiography in the CR Group

One patient developed new anterior wall motion abnormalities after prior revascularization and was thus referred for further testing and management and excluded from this study. There were no other instances of inducible myocardial ischemia or hemodynamic derangements on any stress echocardiogram, which were all diagnostic according to guideline criteria. When compared with baseline, the CR patients achieved a significant improvement in total exercise time (7.5 ± 2.3 vs. 9.5 ± 2.2 min, *p* < 0.001) and METs (8.9 ± 2.4 vs. 10.8 ± 2.5, *p* < 0.001).

## 4. Discussion

The FIT-TWIST study prospectively evaluated the effects of a 36-session CR program on cardiac mechanics in IHD patients who elected to participate in the training program and compared their outcomes with a matched No-CR cohort who did not participate. The salient findings are summarized as follows: (1) when compared with baseline, the CR group experienced significant improvement in LV GLS; (2) when compared with baseline, the No-CR group showed deterioration in RVFWS, with no other changes observed, including in LV GLS; (3) comparisons between CR versus No-CR revealed greater peak LV twist and a healthier RVFWS in CR participants at study completion; and (4) the CR patients achieved a significant improvement in total exercise time and metabolic equivalents of task from baseline to post-CR follow-up stress testing (Graphical Abstract).

### 4.1. LV Myocardial Architecture and Deformational Mechanics

The three-dimensional architecture of the LV is composed of longitudinal subendocardial, circumferential mid-wall, and longitudinally oblique subepicardial myofibers arranged in an ellipsoid helix [20]. GLS is highly reflective of longitudinal subendocardial function, which is the most sensitive layer to ischemic damage, and accounts for approximately 33% of LV stroke volume with a normal value of <−16% [7,21]. Given that LVEF is often preserved in the setting of subendocardial dysfunction due to its reliance on circumferential and radial mechanics, chamber geometry, and loading conditions, GLS outperforms LVEF in detecting subclinical LV systolic dysfunction and in risk stratification [17,22]. Torsional deformation quantified as peak twist around the LV long axis is the result of longitudinal–circumferential fiber shearing forces, with the rotational angle driven by the oblique subepicardial layer [23]. This creates the necessary intra-cavitary pressure for systolic ejection via the minimum required fiber shortening and efficient myocardial energetics, with normal values reported to measure between 14 and 20 degrees [24]. Thus, GLS and LV twist assessment is postulated to represent a transmural global performance index of the LV.

### 4.2. Impact of CR on LV GLS and Peak Twist

The approximately 10% relative increase in GLS in the CR group is consistent with accepted thresholds for meaningful change in LV longitudinal deformation, and more specifically correlates with improvement in subendocardial function [17]. The resultant low normalization of LV GLS to −16.2% with CR is also reassuring for stability of ventricular geometry, as an LV GLS > −15% after acute myocardial infarction has proven predictive of LV dilatation, adverse remodeling, and poorer outcomes at short to mid-term follow-up (*p* < 0.001) [7,25]. Furthermore, LV GLS is established as a superior prognostic marker and a strong independent parameter for the detection of subclinical coronary artery disease, and its observed improvement may reflect exercise-induced disease stabilization [7,26]. Sustained LV twist and torsional mechanics in this setting are also associated with better systolic function and less ventricular remodeling, the latter of which supports fiber orientation and preserves longitudinal–circumferential fiber shearing angles [23,27,28]. Thus, the significantly greater peak LV twist at follow-up amongst CR patients is suggestive of improved transmural mechanical coupling and shearing forces.

Exercise training in previously healthy sedentary individuals has been shown to result in increased LV diastolic volume, peak twist, and functional capacity, which was mirrored in the CR group [29,30]. The increases in average exercise time by 2 min and METs by 2 units in the CR group is commendable and itself portends a good prognosis [4,5]. It should be noted that LV twist is increased by preload and inotropy, and the increase in LV end-diastolic volume, systolic function and mechanics, and functional capacity serve as established surrogates for these parameters [31]. Placed within this context, the improved LV GLS in the CR group, coupled with a significantly better peak twist at follow-up when compared with the No-CR patients, provides insight into global functional reverse remodeling in response to structured exercise training.

### 4.3. RV Mechanics and Impact of CR on RVFWS

The RV free wall is composed of a thin circumferentially oriented outer myocardial layer and a predominant inner layer of longitudinal fibers, which account for approximately 75% of RV systolic contraction and can be assessed by speckle-tracking RVFWS [32]. RV mechanics are vital prognostic markers in IHD that reflect intrinsic contractility and afterload effects from LV systolic dysfunction and elevated filling pressures [33]. While a small cross-sectional study in post-revascularization IHD patients reported no change in standard RV functional assessment after a limited 5-session CR program, data regarding the impact of structured CR programs on RV functional remodeling and mechanics are lacking [34]. Our observations of a decline in RVFWS in the No-CR group, despite a better baseline value, contrasts sharply with the CR group’s post-intervention intragroup stability and better function compared with No-CR at follow-up. It is established that RV performance in IHD is important for functional capacity independent of LV function (regression coefficient 0.73, *p* = 0.02) [35]. This suggests a uniquely protective role in RV performance with structured CR in IHD that requires further investigation.

### 4.4. FIT-TWIST and Prior Studies on CR in IHD

We hypothesize that the type of CR applied to the ischemic heart may modulate beneficial functional reverse remodeling and cardiac mechanics. In a randomized trial of 52 IHD patients post-revascularization assigned to 5-week aerobic CR versus No-CR, a better LV GLS at follow-up was achieved with CR (−15.1 ± 3.7 vs. −12.5 ± 3.6%, *p* < 0.001) [9]. In a similar prospective study of 55 IHD patients, a better LV GLS was achieved after 5 weeks of aerobic plus calisthenics CR when compared with No-CR (−20.1 ± 3.2 vs. −17.4 ± 3.1%, *p* < 0.05) [8]. Conversely, two small randomized trials of 12-week high-intensity interval training and a third prospective observational study of aerobic plus resistance training showed no change or benefit in LV deformational parameters in IHD [10,11,12]. As mentioned previously, the heterogeneity amongst studies regarding CR regimens and length of training does not allow for definitive conclusions. However, the present study supports the reported beneficial impact of aerobic CR on LV longitudinal mechanics, adds to the foundational evidence for its possible role in functional reverse remodeling of LV and RV deformation, and does so on the background of modern guideline-directed medical therapy and coronary revascularization strategies. The prospective study design, strict methodological construct according to societal guidelines allowing for external application of the CR program, and length of training are important strengths of FIT-TWIST. Additionally, the inclusion of LV torsional and RV deformational analysis is novel and, alongside LV GLS, represents a more in-depth analysis of global bi-ventricular performance, which is lacking in prior investigations.

### 4.5. Exercise Training in Patients with Stable IHD

Completion of a CR program primes the patient with IHD to implement exercise training as part of a healthy lifestyle, as physical inactivity is a risk factor for development of de novo or progression of existing coronary artery disease. The benefits of exercise training on myocardial perfusion and metabolism from a mechanistic standpoint include improvement in endothelial function, stabilization of vessel plaque, vasculogenesis and coronary artery collateralization, and regulated platelet activation [36]. It is plausible that these physiologic improvements are reflected in the optimized myocardial mechanics reported in studies of patients completing CR. Importantly, the American Heart Association/American College of Cardiology provide level 1 guideline recommendations for 150 min/week of moderate-intensity or 75 min/week of high-intensity aerobic exercise, and 2 days/week of resistance strength training for patients with IHD, to improve functional capacity, enhance quality of life, decrease hospital admissions, and impart cardiovascular protection [37].

### 4.6. Study Limitations

There are several important limitations that are paramount to discuss when interpreting the FIT-TWIST results. Firstly, this study was a single-center study and the sample size was small. This imparts a selection bias and may increase the tendency of type II statistical error. Additionally, there are several caveats inherent to the propensity-matching modeling utilized. This includes unmeasured variable confounding, loss of sample size and power due to unmatched subjects, and the interplay between covariate balancing and causal validity, all of which may limit generalizability. Finally, because the echocardiographic measurements were performed in an unblinded manner, there is a potential susceptibility to observer or detection bias. Secondly, most of the patients enrolled were male, tempering the applicability of the findings to females given the known differences in cardiac remodeling between them [38]. Thirdly, the study cohort consisted of stable IHD patients across the spectrum of coronary syndromes. While there was no difference between the CR and No-CR groups in the severity of obstructive coronary artery disease or prior myocardial infarction by standard criteria, the extent of myocardial damage or scarring was not identified or assessed by advanced techniques such as myocardial perfusion imaging or coronary flow reserve. Myocardial scarring limits the reserve the cardiac chambers have for reverse remodeling, and its potential impact in the present study is unknown [2,3]. Fourthly, the patients who completed < 80% of their CR sessions represent a form of attrition bias and introduce a form of uncontrollable confounding. Additionally, whether subjective parameters such as motivation or persistent symptomatology impacted CR participation or outcomes is unknown. Fifthly, the speckle-tracking analyses of cardiac mechanics were performed on a single vendor software package (EchoPAC v206, General Electric Healthcare, Waukesha, WI, USA). While there have been large strides in intervendor standardization of cardiac deformation measures over the last decade by societal and industry task forces, important variability persists which precludes strict application of the results outside of the methods described herein. Finally, while less dependent than traditional measures of ventricular function such as ejection fraction, loading conditions do impact measures of cardiac deformation and, in particular, longitudinal mechanics such as LV GLS and RVFWS [7]. Parameters such as myocardial work, which integrate the degree of myocardial longitudinal shortening with systemic blood pressure to provide a measure of myocardial energy expenditure and efficiency, were not measured and remain under investigation for clinical use [39]. Notably, both groups had similar rates of hypertension, anti-hypertensive medication use, and measures of systolic and diastolic blood pressure, which mitigates potential intergroup discrepancies.

## 5. Conclusions

In conclusion, the FIT-TWIST study prospectively evaluated the effects of a guideline-oriented 36-session CR program using aerobics on cardiac mechanics in IHD patients. The significant improvement in LV GLS amongst CR patients and their better follow-up peak twist when compared with the No-CR group suggests that structured CR positively influences transmural LV myocardial deformational mechanics in patients with IHD and may further serve a uniquely protective role in RV performance. FIT-TWIST adds to the foundational evidence for the possible role of CR in functional reverse remodeling of cardiac mechanics and does so on the background of modern medical and revascularization strategies. Future studies with larger diverse cohorts and external validation of the findings are eagerly encouraged.

## Figures and Tables

**Table 1:** Baseline characteristics according to participation in cardiac rehabilitation.

Variable	Cardiac Rehabilitation	No Cardiac Rehabilitation	*p*-Value
Age	62 ± 9	63 ± 10	0.679
Body surface area	1.96 ± 0.18	1.98 ± 0.27	0.735
Heart rate (beats/minute)	65 ± 10	71 ± 12	0.044
Systolic blood pressure (mmHg)	126 ± 18	135 ± 25	0.172
Diastolic blood pressure (mmHg)	75 ± 12	79 ± 14	0.279
Male	22 (82%)	22 (82%)	1
Smoking	7 (26%)	11 (41%)	0.248
Hypertension	20 (74%)	20 (74%)	1
Diabetes mellitus	10 (37%)	6 (22%)	0.233
Dyslipidemia	20 (74%)	14 (52%)	0.091
Atrial fibrillation	3 (11%)	5 (19%)	0.704
Peripheral Vascular Disease	1 (4%)	2 (7%)	1
Coronary artery disease			
ST-elevation myocardial infarction	12 (44%)	11 (41%)	0.783
Non-ST-elevation myocardial infarction	10 (37%)	12 (44%)	0.580
Unstable angina	2 (7%)	4 (15%)	0.669
Stable angina	3 (11%)	0	0.236
Anterior myocardial infarction	13 (48%)	11 (41%)	0.584
Left main coronary artery disease	2 (7%)	5 (19%)	0.420
Left anterior descending artery disease	24 (89%)	24 (89%)	1
3-vessel coronary artery disease	9 (33%)	8 (30%)	0.770
Coronary revascularization			
Percutaneous coronary intervention	18 (67%)	23 (85%)	0.111
Coronary artery bypass graft surgery	8 (30%)	4 (15%)	0.327
Medical therapy	1 (3%)	0	1
Medications			
Aspirin	25 (93%)	26 (96%)	0.552
P2Y12-inhibitor	21 (78%)	25 (93%)	0.125
Beta-blocker	25 (93%)	22 (82%)	0.224
Angiotensin converting enzyme	17 (63%)	17 (63%)	1
Inhibitor/receptor blocker			
Statin	27 (100%)	26 (96%)	0.313
Novel oral anticoagulant	4 (15%)	4 (15%)	1

**Table 2:** Two-dimensional and speckle-tracking echocardiographic analyses at baseline and follow-up according to participation in cardiac rehabilitation.

	Baseline Assessment			Follow-Up Assessment		
Variable	Cardiac Rehabilitation	No Cardiac Rehabilitation	*p*-Value	Cardiac Rehabilitation	No Cardiac Rehabilitation	*p*-Value
2-dimensional and Doppler echocardiography						
LV ejection fraction (%)	52 ± 7 ^a^	52 ± 9	0.904	55 ± 8 ^a^	54 ± 9	0.639
LV end-diastolic volume index (mL/m^2^)	55 ± 23	50 ± 19	0.420	56 ± 17	47 ± 16	0.040
LV end-systolic volume index (mL/m^2^)	26 ± 15	25 ± 13	0.669	25 ± 11	22 ± 12	0.278
LV internal diastolic diameter index (mm/m^2^)	25 ± 5	25 ± 3	0.842	25 ± 5	24 ± 3	0.052
LV internal systolic diameter index (mm/m^2^)	19 ± 4	18 ± 4	0.432	18 ± 3	17 ± 4	0.284
Interventricular septal thickness (mm)	10 ± 2	11 ± 2	0.255	10 ± 1	13 ± 2	0.213
Posterior wall thickness (mm)	10 ± 1	11 ± 2	0.723	10 ± 1	11 ± 2	0.449
Transmitral E-wave velocity (m/s)	0.71 ± 0.14	0.74 ± 0.2	0.542	0.67 ± 0.16	0.68 ± 0.19	0.866
Transmitral E/A wave ratio	1.3 ± 0.7	1.2 ± 0.7	0.623	1.1 ± 0.6	1 ± 0.4	0.544
E/e’ ratio	10 ± 3 ^b^	10 ± 3	0.779	9 ± 3 ^b^	10 ± 2	0.282
Left atrial volume index (mL/m^2^)	30 ± 9	30 ± 7	0.823	28 ± 7	29 ± 10	0.762
Right ventricular basal diameter (mm)	35 ± 3	36 ± 5	0.832	35 ± 4	36 ± 6	0.286
Tricuspid annular plane systolic excursion (mm)	17 ± 4	17 ± 5	0.598	17 ± 4	16 ± 6	0.402
Speckle-tracking echocardiography						
LV global longitudinal strain (%)	−14.9 ± 2.9 ^c^	−14.8 ± 3.1	0.947	−16.2 ± 3.1 ^c^	−15 ± 3.3	0.175
Peak LV twist (degrees)	14.4 ± 7.4	12.2 ± 6.9	0.266	16.8 ± 5.3	12.1 ± 4.2	0.001
Time to peak LV twist (ms)	343 ± 59	332 ± 93	0.619	355 ± 57	348 ± 62	0.685
Time to peak LV untwist (ms)	988 ± 128	931 ± 192	0.211	978 ± 160	901 ± 162	0.085
Right ventricular free wall strain (%)	−20.4 ± 4.3	−22.9 ± 4.6 ^d^	0.045	−22.2 ± 4.5	−19.3 ± 5.4 ^d^	0.044

LV = left ventricle. Intra-group repeated measure paired *t*-test: ^a^ *p* = 0.010; ^b^ *p* = 0.032; ^c^ *p* = 0.003; ^d^ *p* = 0.009.

## Data Availability

Due to institutional IRB rules and regulations on data handling, the supporting research data is not available.

## Supplementary Materials

The following supporting information can be downloaded at https://www.mdpi.com/article/10.3390/jcdd13020062/s1, Table S1: Two-dimensional and speckle-tracking echocardiographic analyses at baseline and follow-up in patients participating in cardiac rehabilitation; Table S2: Two-dimensional and speckle-tracking echocardiographic analyses at baseline and follow-up in patients not participating in cardiac rehabilitation.

## Abbreviations

CR	cardiac rehabilitation
ECG	electrocardiogram
FIT-TWIST	Impact of Fitness on Cardiac Torsion and Wall Mechanics in Ischemic Heart Disease Study
GLS	global longitudinal strain
IHD	ischemic heart disease
LV	left ventricle
LVEF	left ventricular ejection fraction
METs	metabolic equivalents of task
No-CR	no cardiac rehabilitation
RV	right ventricle
RVFWS	right ventricular free wall strain
SD	standard deviation
