# Supplementary material for: Impact of Fitness on Cardiac Torsion and Wall Mechanics in Ischemic Heart Disease Study (FIT-TWIST)
Source: J Cardiovasc Dev Dis. 2026 Jan 24;13(2):62. doi: 10.3390/jcdd13020062 (PMC12942191; doi:10.3390/jcdd13020062)
Supplement: Supplementary file 1 [file jcdd-13-00062-s001.zip › jcdd-4087868-supplementary.pdf]

Supplementary Table S1. Two-dimensional and speckle-tracking echocardiographic analyses at baseline and follow-up in patients participating in cardiac rehabilitation.

Variable	Baseline CR	Follow-Up CR	P-value
2-dimensional and Doppler echocardiography			
LV ejection fraction (%)	52 ± 7	55 ± 8	0.010
LV end-diastolic volume index (ml/m^2)	55 ± 23	56 ± 17	0.564
LV end-systolic volume index (ml/m^2)	26 ± 15	25 ± 11	0.644
LV internal diastolic diameter index (mm/m^2)	25 ± 5	25 ± 5	0.435
LV internal systolic diameter index (mm/m^2)	19 ± 4	18 ± 3	0.270
Interventricular septal thickness (mm)	10 ± 2	10 ± 1	0.355
Posterior wall thickness (mm)	10 ± 1	10 ± 1	0.472
Transmitral E-wave velocity (m/s)	0.71 ± 0.14	0.67 ± 0.16	0.406
Transmitral E/A wave ratio	1.3 ± 0.7	1.1 ± 0.6	0.073
E/e’ ratio	10 ± 3	9 ± 3	0.032
Left atrial volume index (ml/m^2)	30 ± 9	28 ± 7	0.150
Right ventricular basal diameter (mm)	35 ± 3	35 ± 4	0.344
Tricuspid annular plane systolic excursion (mm)	17 ± 4	17 ± 4	0.562
Speckle-tracking echocardiography			
LV global longitudinal strain (%)	-14.9 ± 2.9	-16.2 ± 3.1	0.003
Peak LV twist (degrees)	14.4 ± 7.4	16.8 ± 5.3	0.162
Time to peak LV twist (ms)	343 ± 59	355 ± 57	0.450
Time to peak LV untwist (ms)	988 ± 128	978 ± 160	0.757
Right ventricular free wall strain (%)	-20.4 ± 4.3	-22.2 ± 4.5	0.112

CR=cardiac rehabilitation; LV=left ventricle.

Supplementary Table S2. Two-dimensional and speckle-tracking echocardiographic analyses at baseline and follow-up in patients not participating in cardiac rehabilitation.

Variable	Baseline No-CR	Follow-Up No-CR	P-value
2-dimensional and Doppler echocardiography			
LV ejection fraction (%)	52 ± 9	54 ± 9	0.130
LV end-diastolic volume index (ml/m^2)	50 ± 19	47 ± 16	0.291
LV end-systolic volume index (ml/m^2)	25 ± 13	22 ± 12	0.114
LV internal diastolic diameter index (mm/m^2)	25 ± 3	24 ± 3	0.296
LV internal systolic diameter index (mm/m^2)	18 ± 4	17 ± 4	0.348
Interventricular septal thickness (mm)	11 ± 2	13 ± 2	0.423
Posterior wall thickness (mm)	11 ± 2	11 ± 2	0.838
Transmitral E-wave velocity (m/s)	0.74 ± 0.2	0.68 ± 0.19	0.214
Transmitral E/A wave ratio	1.2 ± 0.7	1 ± 0.4	0.086
E/e’ ratio	10 ± 3	10 ± 2	0.303
Left atrial volume index (ml/m^2)	30 ± 7	29 ± 10	0.368
Right ventricular basal diameter (mm)	36 ± 5	36 ± 6	0.512
Tricuspid annular plane systolic excursion (mm)	17 ± 5	16 ± 6	0.218
Speckle-tracking echocardiography			
LV global longitudinal strain (%)	-14.8 ± 3.1	-15 ± 3.3	0.831
Peak LV twist (degrees)	12.2 ± 6.9	12.1 ± 4.2	0.946
Time to peak LV twist (ms)	332 ± 93	348 ± 62	0.364
Time to peak LV untwist (ms)	931 ± 192	901 ± 162	0.495
Right ventricular free wall strain (%)	-22.9 ± 4.6	-19.3 ± 5.4	0.009

No-CR=no cardiac rehabilitation; LV=left ventricle.
